# Genome sequence of *Erinnyis ello granulovirus* (ErelGV), a natural cassava hornworm pesticide and the first sequenced sphingid-infecting betabaculovirus

**DOI:** 10.1186/1471-2164-15-856

**Published:** 2014-10-04

**Authors:** Daniel Mendes Pereira Ardisson-Araújo, Fernando Lucas de Melo, Miguel de Souza Andrade, William Sihler, Sonia Nair Báo, Bergmann Morais Ribeiro, Marlinda Lobo de Souza

**Affiliations:** Cell Biology Department, Laboratory of Baculovirus, University of Brasília, 70910-900 Brasília, DF Brazil; Embrapa Genetic Resources and Biotechnology, Biological Station Park, 70770-917 Brasília, DF Brazil

**Keywords:** Biological control, Cassava hornworm, Baculovirus, Sphingidae, Horizontal gene transfer, *Betabaculovirus* evolution

## Abstract

**Background:**

Cassava (*Manihot esculenta*) is the basic source for dietary energy of 500 million people in the world. In Brazil, *Erinnyis ello ello* (Lepidoptera: Sphingidae) is a major pest of cassava crops and a bottleneck for its production. In the 1980s, a naturally occurring baculovirus was isolated from *E. ello* larva and successfully applied as a bio-pesticide in the field. Here, we described the structure, the complete genome sequence, and the phylogenetic relationships of the first sphingid-infecting betabaculovirus.

**Results:**

The baculovirus isolated from the cassava hornworm cadavers is a betabaculovirus designated *Erinnyis ello granulovirus* (ErelGV). The 102,759 bp long genome has a G + C content of 38.7%. We found 130 putative ORFs coding for polypeptides of at least 50 amino acid residues. Only eight genes were found to be unique. ErelGV is closely related to ChocGV and PiraGV isolates. We did not find typical homologous regions and *cathepsin* and *chitinase* homologous genes are lacked. The presence of *he65* and *p43* homologous genes suggests horizontal gene transfer from *Alphabaculovirus*. Moreover, we found a nucleotide metabolism-related gene and two genes that could be acquired probably from *Densovirus*.

**Conclusions:**

The ErelGV represents a new virus species from the genus *Betabaculovirus* and is the closest relative of ChocGV. It contains a *dUTPase*-like, a *he65*-like, *p43*-like genes, which are also found in several other alpha- and betabaculovirus genomes, and two *Densovirus*-related genes. Importantly, recombination events between insect viruses from unrelated families and genera might drive baculovirus genomic evolution.

**Electronic supplementary material:**

The online version of this article (doi:10.1186/1471-2164-15-856) contains supplementary material, which is available to authorized users.

## Background

Cassava (*Manihot esculenta*) is the basic source for dietary energy of 500 million people in tropical and subtropical areas of Africa, Asia, and Latin America [1]. In Brazil, the hornworm *Erinnyis ello ello* (Lepidoptera: Sphingidae) is one of the most important pests [2] occurring throughout the year and greatly impacting cassava production [3, 4]. This pest has been observed in 35 plant species, especially in the Euphorbiaceae family [5, 6]. In large infestations, the cassava pest may reduce by 50% the roots yield. In the 1980s, a naturally occurring baculovirus was isolated from this pest and applied as a bio-pesticide in Brazil [5]. The biological control program has proven to be safe and economical [5, 6]. However, genomic and structural information about this virus is lacking.

The *Baculoviridae* is a family of insect viruses with circular double-stranded genomic DNA [7–9] that have been successfully applied for the control of agricultural and forest pests [10]. So far, *Alpha* and *Betabaculovirus* are the most studied baculovirus genera; both infect Lepidoptera [7]. The infection is initiated when larvae feed on foliage contaminated with orally infectious occlusion bodies (OBs) [11] that release occlusion derived-virions (ODVs) in the midgut [12]. Early after primary midgut epithelial cell infection, budded virions (BV) are produced and cause systemic infection. Infection symptoms include cuticle discoloration, movement loss, and incapability for feeding [13, 14].

Few full-length betabaculovius genome sequences are available compared to those from *Alphabaculovirus* and none of them was isolated from sphingid host. In this context, identification and sequencing of virus species from different lepidopteran families will provide a wider empirical database to help understand baculovirus evolution [15, 16]. Here, we presented the morphological characterization, the complete genome sequence, and the phylogenetic analyses of the natural cassava hornworm pesticide, the first completely sequenced betabaculovirus isolated from a sphingid host.

## Results and discussion

### Virus characterization and genome features

**Figure 1:**
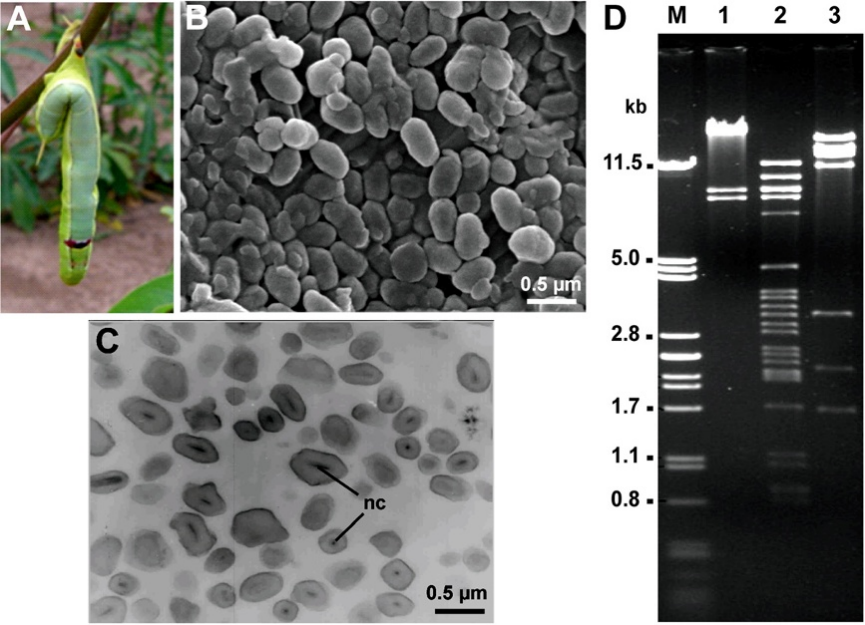
***Erinnyis ello granulovirus*** **(ErelGV) infection and virus characterization. (A)** Cassava hornworm cadaver found hanging in the field due to terminal baculovirus infection (Source: José Osmar Lorenzi). **(B)** Scanning and **(C)** transmission electron micrographs reveal granular occlusion bodies containing singly embedded rod-shaped nucleocapsid (nc) (scale bars = 0.5 μm). **(C)** Restriction enzyme profile of Brazilian isolate genomic DNA. Agarose gel electrophoresis-resolved DNA fragments digested with *Hin*dIII (lane 1), *Eco*RI (lane 2), *Bam*HI (lane 3).

A naturally occurring baculovirus was isolated from dead cassava hornworm (*E. ello ello*) caterpillars in crops from the South of Brazil in 1986. As shown in Figure 1A, the larvae is usually found hanged in cassava apical leaves, which is a characteristic symptom of the baculovirus infections [17]. Neither cuticle melanization nor post-mortem melting phenotypes were observed among the caterpillar cadavers, an attribute which probably facilitated virus collection and use for pesticide production as previously observed in another baculovirus (*Anticarsia gemmatalis multiple nucleopolyhedrovirus* - AgMNPV) [10]. Ultrastructural analyses revealed a granular OB with irregular form and size (Figure 1B) containing single rod-shaped nucleocapsid (Figure 1C). Both of these structural features, *i.e.* granular form and nucleocapsid shape, are typical of viruses from the genus *Betabaculovirus* [8, 18] and thus, we named it *Erinnyis ello granulovirus* (ErelGV) isolate Br-S86 (Brazil/South/1986). Two other cassava hornworm-isolated granuloviruses were previously reported, one isolated in Colombia [19] and another from an undisclosed geographical source [20]. Restriction endonuclease profile analyses (Figure 1D) suggest that the Brazilian and the Colombian viruses (previously published in [19]) are either variants of the same species or are distinct species infecting the same host. However, the absence of sequence data from the latter prevents establishment of any phylogenetic relationship.

We sequenced the genome of ErelGV, the first completely sequenced sphingid host-isolated betabaculovirus (Genbank accession number KJ406702). The genome is 102,759 bp long with a G + C content of 38.7% (Table 1). We found 130 putative genes coding for polypeptides of at least 50 amino acid residues. Additional file 1: Table S1 summarizes the ErelGV genes and compares each predicted protein sequence with its orthologs in other baculoviruses. Eight of these were shown to be unique (*ErelOrf-11*, *ErelOrf-15*, *ErelOrf-27*, *ErelOrf-53*, *ErelOrf-59*, *ErelOrf-70*, *ErelOrf-90*, *ErelOrf-102*), and all of them are peptides with no significant similarity to any other sequence in GenBank. All 37 *Baculoviridae* core genes were found and no typical homologous regions (hrs) were detected. However, we identified five putative homologous regions (hrs)/repeat regions lacking typical alphabaculovirus hr palindromes. This feature is also found in both *Choristoneura occidentalis granulovirus* (ChocGV) and *Pieris rapae granulovirus* (PiraGV) genomes. As observed in ChocGV [21], ErelGV lacks both *gp37* and *exon0*, which was previously predicted for being shared among all *Alpha* and *Betabaculovirus* [22].

**Table 1:** **All species from the genus** ***Betabaculovirus*** **completely sequenced to date**

Virus species	Host Family	Size (bp)	ORFs	Accession	Refs.
*Adoxophyes orana granulovirus*	Tortricidae	99,657	119	AF547984	[23]
*Agrotis segetum granulovirus* Xinjiang	Noctuidae	131,680	132	AY522332	[24]
*Agrotis segetum granulovirus* L1	Noctuidae	131,442	149	KC994902	[24]
*Choristoneura occidentalis granulovirus*	Tortricidae	104,710	116	DQ333351	[21]
*Clostera anachoreta granulovirus*	Notodontidae	101,487	123	HQ116624	[25]
*Clostera anastomosis L. granulovirus*	Notodontidae	101,818	123	KC179784	u/d
*Cryptophlebia leucotreta granulovirus*	Tortricidae	110,907	129	AY229987	[26]
*Cydia pomonella granulovirus*	Tortricidae	123,500	143	U53466	[27]
*Epinotia aporema granulovirus*	Tortricidae	119,092	132	JN408834	[28]
*Erinnyis ello granulovirus*	Sphingidae	102,759	135	KJ406702	**-**
*Helicoverpa armigera granulovirus*	Noctuidae	169,794	179	EU255577	[29]
*Phthorimaea operculella granulovirus*	Gelechiidae	119,217	130	AF499596	u/d
*Pieris rapae granulovirus* China	Pieridae	108,592	120	GQ884143	[30]
*Pieris rapae granulovirus* E3	Pieridae	108,476	125	GU111736	u/d
*Pieris rapae granulovirus* South Korea	Pieridae	108,658	120	JX968491	u/d
*Plutella xylostella granulovirus*	Plutellidae	100,999	120	AF270937	[31]
*Pseudaletia unipuncta granulovirus*	Noctuidae	176,677	183	EU678671	u/d
*Spodoptera litura granulovirus*	Noctuidae	124,121	136	DQ288858	[32]
*Xestia c-nigrum granulovirus*	Noctuidae	178,733	181	AF162221	[33]

u/d - unpublished data.

### Phylogenetic analysis

**Figure 2:**
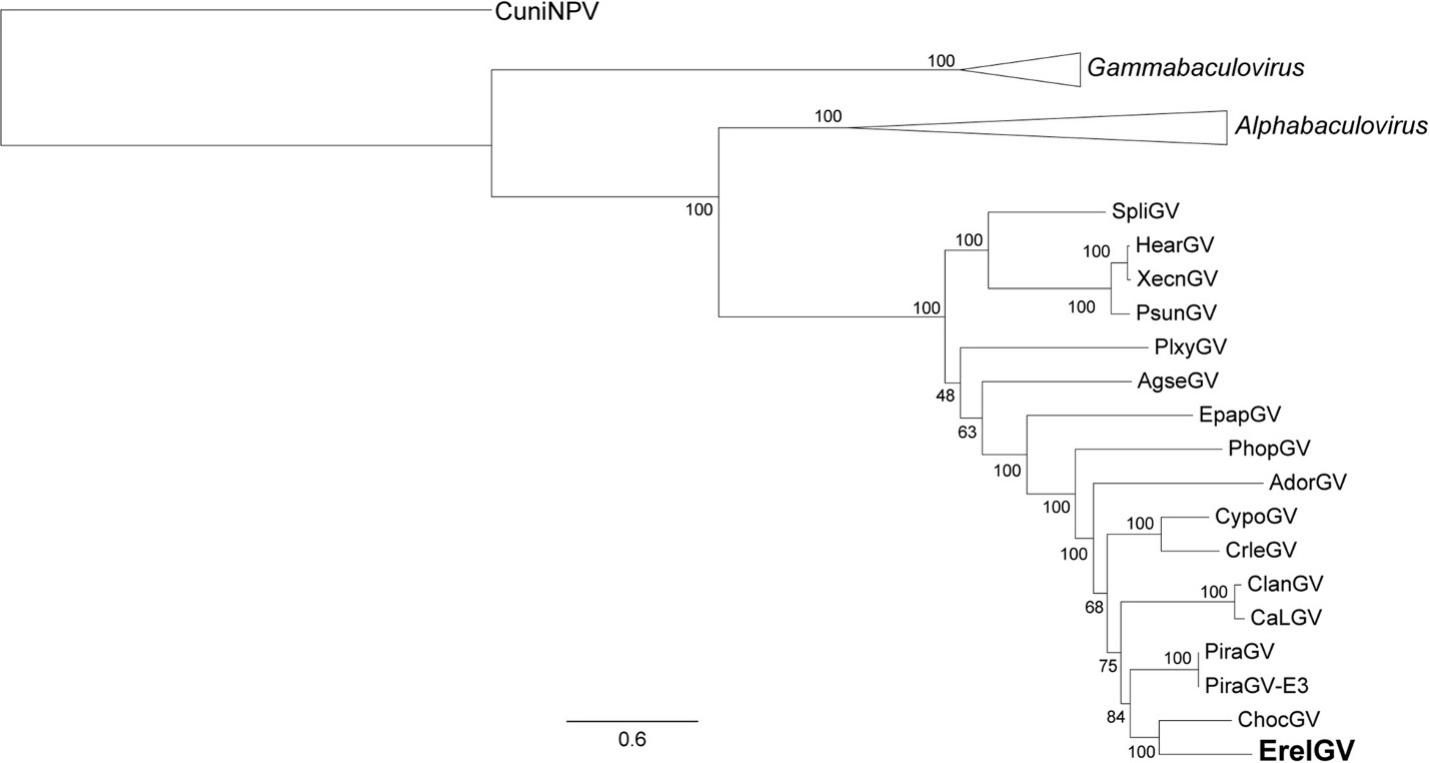
**Maximum likelihood tree for** ***Betabaculovirus*** **.** The phylogenetic inference was based on the concatenated amino acid sequences of the 37 core genes identified in all complete baculovirus genome sequences. We collapsed all the *Gammabaculovirus* and *Alphaphabaculovirus*. The CuniNPV was used as root. ErelGV (boldface) clustered with ChocGV and both were closely related to PiraGV isolates.

In order to better understand the evolutionary history of ErelGV and the genus *Betabaculovirus*, we carried out a maximum likelihood phylogenetic analysis using the 37 baculovirus core gene alignment from all baculovirus genome available. ErelGV clustered with ChocGV and both viruses share the same ancestor with PiraGV isolates (Figure 2). Since the Chinese and Korean PiraGV isolates are very similar to each other (99.5%), we have included only the Chinese isolate in our analyses. Using Mauve alignment [34], we found that ChocGV and PiraGV genomes have respectively 38.5% and 34.5% of global pairwise identity when compared to ErelGV genome. Additionally, our phylogenetic analyses did not find support for *Betabaculovirus* division in two clades (A and B), as described previously using neighbor joining clustering method [25, 28]. Phylogenetic relationships in *Baculoviridae*, in particular in the genus *Betabaculovirus*, are difficult to discern due to the limited number of sequenced genomes available (Table 1). Therefore, we further evaluated ErelGV phylogenetic relationships using *granulin*, *lef-8*, and *lef-9* partial gene dataset as previously carried out [20, 35] (28 partial sequences), but including new sequences publicly available (seven sequences from completely sequenced baculovirus) totalizing 35 granulovirus sequences. This analysis revealed that ErelGV isolate Br-S86 is closely related to another ErelGV (also called EeGV) from the Steinhaus collection [20] and that both are closer to *Andraca bipunctata granulovirus* (AnbiGV) (data not shown).

### *Betabaculovirus*gene comparison

**Figure 3:**
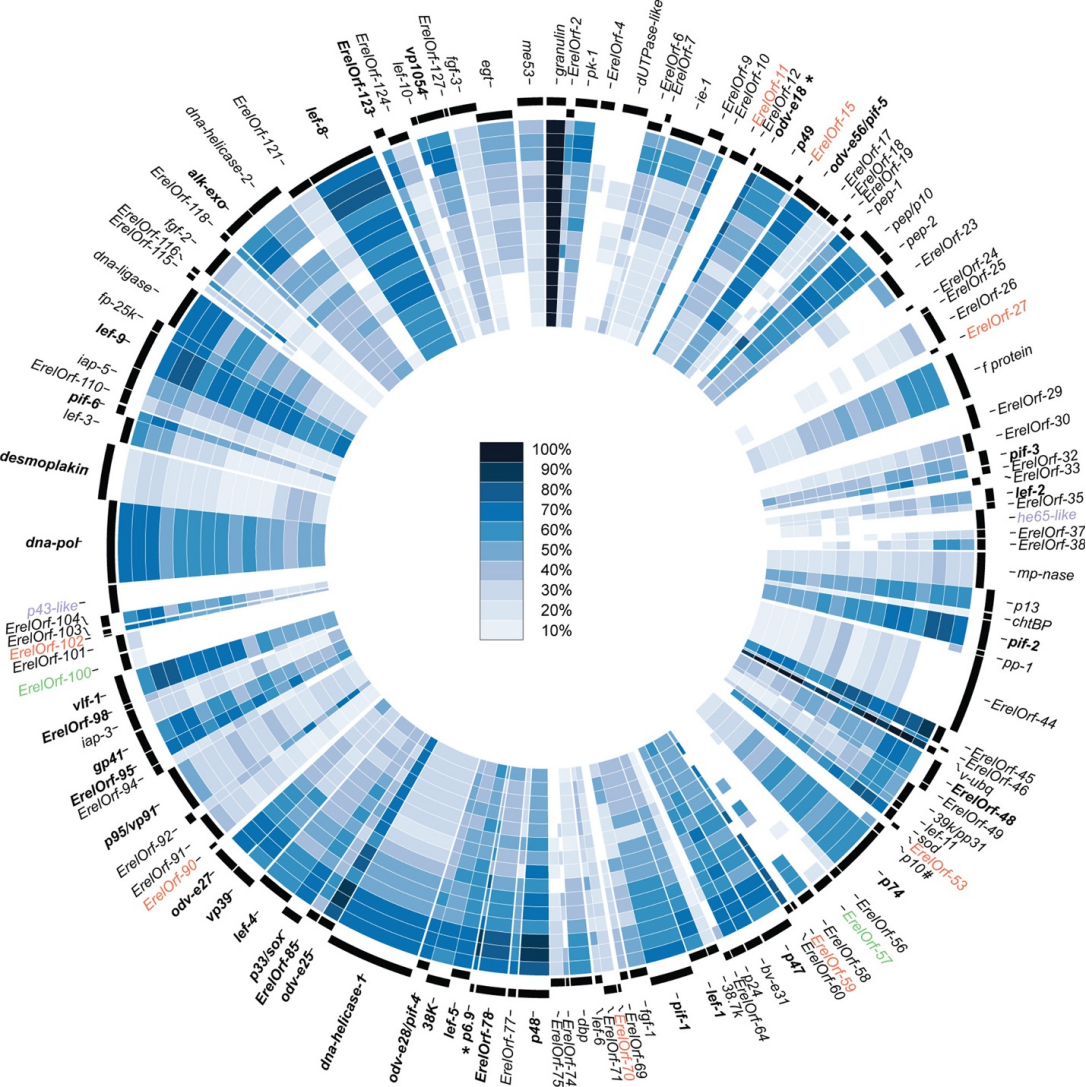
**Gene comparison of ErelGV genome and all completely sequenced betabaculoviruses available in Genbank.** CDS identities were acquired by BLAST analysis and ranked from 0 to 100%. From the outermost ring: ChocGV, PiraGV-E3, PiraGV-China, ClanGV, CaLGV, CrleGV, CypoGV, AdorGV, PhopGV, EpapGV, AgseGV, PlxyGV, PsunGV, XecnGV, HearGV, and SpliGV-K1. For this representation, gene synteny is not taken into account. CDS that were absent in the ErelGV genome but present in the query sequences were not displayed. To prevent the missing of known homologues, like p6.9 and odv-e18 (asterisk), all the low identity hits (bellow 20%) were plotted as well. Unique genes are shown in red, core genes are in boldface, and *Densovirus*-related genes are shown in green.

We performed BLAST comparisons between ErelGV and all other full betabaculovirus genomes available in Genbank using the CGView Comparison Tool [36] and CIRCOS [37]. As shown in Figure 3, most of the ErelGV-encoded ORFs are conserved among all betabaculovirus, but protein similarity varies widely across the species. Some structural proteins, such as granulin and the *per os* infectivity factors (PIFs), were the most conserved genes. Conversely, F protein, the major *Betabaculovirus* envelope fusion protein (EFP, encoded by *ErelOrf-28*) and matrix metalloproteinase (MMP, a stromelysin-1-like protein, encoded by *ErelOrf-39*) were particularly variable despite of both being present in every betabaculovirus sequenced to date. The EFP is essential for cell-to-cell movement and systemic virus spread [7]. GP64 is the EFP found in Group I *Alphabaculovirus* and all orthologs are closely related to each other (81% of protein sequence identity), whereas the F protein, found in both *Alpha* and *Betabaculovirus* [38], is very diverse (20 to 40% sequence identity). Interestingly, deletion of the *gp64* or *f protein* genes is lethal for BV propagation in *Autographa californica multiple nucleopolyhedrovirus* (AcMNPV) [39] and *Helicoverpa armigera nuclepolyhedrovirus* (HaNPV) [40, 41], respectively. The deficiency can be rescued by *efp* homologs from many different viruses in the case of AcMNPV [42], but the opposite is not true; AcMNPV *gp64* is not able to completely rescue an *efp*-deleted HaNPV. However, it is not clear why the F protein from *Plutella xylostella granulovirus* (PlxyGV) is not able to rescue the infectivity of *gp64*-null AcMNPV [42] but that from AgseGV can. PlxyGV causes systemic infection to the diamondback moth *P. xylostella* (Plutellidae) larvae [43] and AgseGV infects the cutworm *A. segetum* (Noctuidae) [44]. Thus, the betabaculovirus EFP variability might reflect the cell machinery adjustment at the insect family level considering that AcMNPV infects caterpillar from the same insect family of *A. segetum*. A second highly variable gene, MMP, is a proteinase able to produce a distinct pattern of melanization in *Bombyx mori* larvae infected with the *Xestia c-nigrum granulovirus* (XecnGV) metalloproteinase-expressing *Bombyx mori nucleopolyhedrovirus* [45]. The enzyme is thought to enhance, replace, or act synergistically with proteins from virus or host playing an important role in the virus spread [46]. This variability is not unexpected since granulovirus genomes vary in content with respect to the presence or absence of the proteases *cathepsin* and *enhancin* genes and also the *chitinase* gene [12, 45–47].

### Lack of *cathepsin*and *chitinase*genes

ErelGV lacks *cathepsin* and *chitinase* genes, despite of their importance for promoting baculovirus horizontal transmission [48]. This feature can explain the integrity of caterpillar flesh and light color after death (Figure 1A). Other betabaculovirus genomes also lack both enzymes: complete deletion in ChocGV [21], *Adoxophyes orana granulovirus* (AdorGV) [23], *Phthorimaea opercullela granulovirus* (PhopGV) (unpublished), PlxyGV [31] and *Spodoptera litura granulovirus* (SpliGV) [32]; *Cryptophlebia leucotreta granulovirus* (CrleGV) [26] *chitinase* has an interruption; and in *Helicoverpa armigera granulovirus* (HearGV) [29] only *cathepsin* is absent. Interestingly, most of these deletions seem to have occurred independently of each other within *Betabaculovirus* (data not shown), aside from ChocGV and ErelGV in which is strongly supported an ancestral lacking. Thus, it is reasonable to expect that AnbiGV, the closest relative to ErelGV, might also lack both *cathepsin* and *chitinase*. Taken together, these results reinforce the notion that both genes are most likely non-essential for the persistence of baculoviruses in the environment. Conversely, previous work from our research team has shown that introduction of *cathepsin* and *chitinase* from *Choristoneura fumiferana defective nucleopolyhedrovirus* into AgMNPV (which naturally lacks both genes) increases pathogenicity and occlusion body production relative to the wild type virus [49].

### *dUTPase-like*gene

*ErelOrf-5* codes for a nucleotide metabolism-related gene homologous to *Orgyia pseudotsugata multiple nucleopolyhedrovirus* (OpMNPV) *Orf-31*. The gene seems to be composed of a fusion between two distinct ORFs; the C-terminal portion is related to a baculovirus *thymidylate kinase*-like gene and the N-terminal portion is related to several *dUTPase*-like genes. The thymidylate kinase enzyme catalyzes a critical step in the biosynthesis of deoxythymidine triphosphate [50]. dUTPase catalyses dUTP dephosphorylation to generate dUMP [51]. High levels of dUTP can be deleterious for virus genomic DNA replication since dTTP can be substituted for dUTP during DNA synthesis [52]. A high dUTP/dTTP ratio promotes uracil incorporation into DNA. Uracils in DNA are then targeted by uracil DNA glycosylase and excised, leading to futile repair cycles and DNA breakage and or translesional DNA synthesis [53, 54]. Nucleotide metabolism-related enzyme acquisition is common in baculoviruses [28] and could avoid this deleterious response by decreasing the dUTP/dTTP ratio, however how these genes alter the virus fitness is not clear [55].

### The *he65*-like and *p43*-like genes

**Figure 4:**
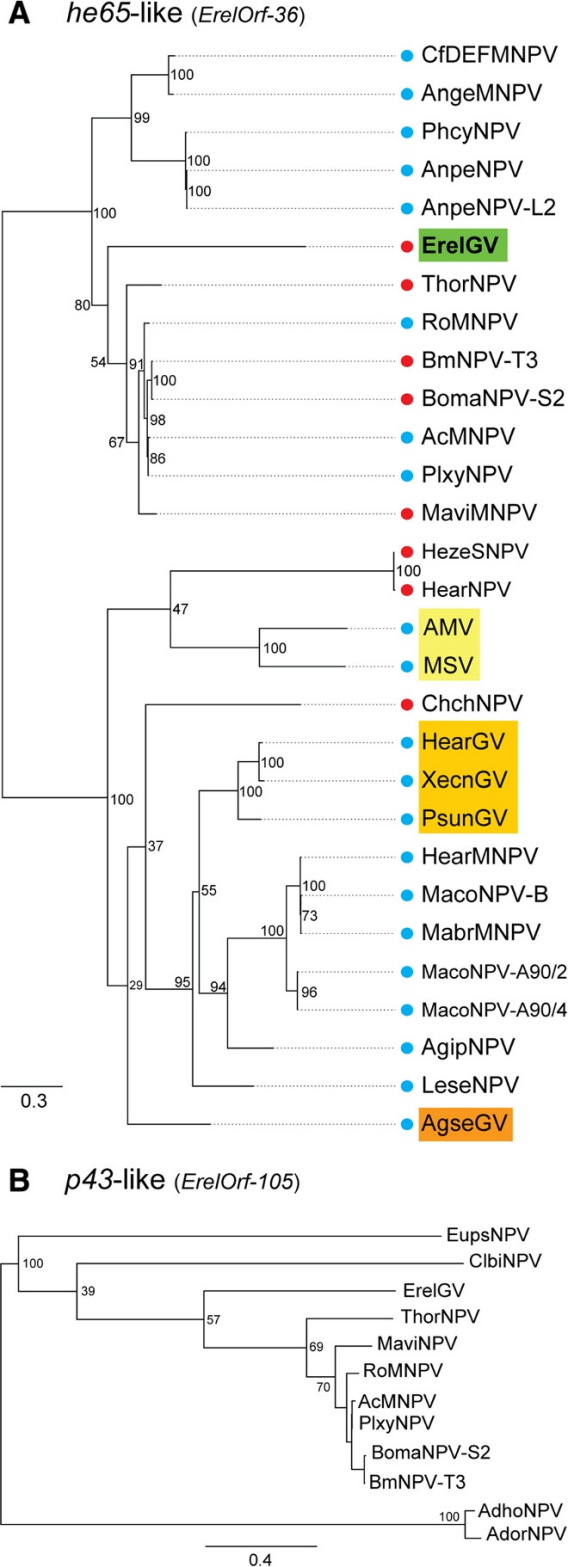
**Phylogeny of** ***he65*** **and** ***p43*** **reveals horizontal gene transfer in ErelGV from** ***Alphabaculovirus*** **. (A)** The maximum likelihood (ML) tree was inferred using the conserved amino-terminal region alignment of *he65*-like gene for 36 baculoviruses and two entomopoxviruses. Circles indicate the presence (blue) or absence (red) of the carboxy-terminal region. The postulated horizontal gene transfer (HGT) events are highlighted for *Betabaculovirus* (light and dark orange), *Betaentomopoxvirus* (yellow), and ErelGV (green). **(B)** ML-Phylogenetic reconstruction for *p43*-like gene found in ErelGV genome. The trees are midpoint rooted for purposes of clarity.

The ErelGV genome contains homologues of the *he65* and *p43* genes. Homologues of *he65* are harbored by several alphabaculoviruses, four betabaculoviruses (*Agrotis segetum granulovirus* (AgseGV), HearGV, *Pseudaletia unipuncta granulovirus* (PsunGV), and *Xestia c-nigrum granulovirus* (XecnGV)), and two betaentomopoxviruses (*Amsacta moorei entomopoxvirus* - AMV and *Mythimna separate entomopoxvirus* - MSV). This gene is a member of a distinct RNA ligase family related to the *T4 RNA ligase gp63*-like gene and is present in all the domains of life (Bacteria, Archaea, and Eukarya) [7, 56]. The alignment of baculovirus and entomopoxvirus *he65*-like genes revealed large, independent, and recurrent deletions in the C-terminal region (data not shown), which contain five nucleotidyl transferase motifs [56]. The amino-terminal region was highly conserved although no previously characterized motifs were present. We performed a phylogenetic reconstruction based on this conserved domain. The *he65* reconstruction revealed distinct horizontal gene transfer (HGT) events from *Alphabaculovirus* to *Betabaculovirus* and *Betaentomopoxvirus* (Figure 4A). *Betabaculovirus* likely endured two independent acquisitions from Group II *Alphabaculovirus* in distinct genomic regions: (*i*) a synapomorphic introduction for HearGV, PsunGV, and XecnGV (Figure 4A, orange rectangle); and (*ii*) an additional gain for AgseGV (Figure 4A, dark orange rectangle). Importantly, support for AgseGV branch is low. However, the genomic context of the gene is conserved among HearGV, PsunGV, and XecnGV but not in AgseGV (data not shown), reinforcing our hypothesis that two independent introductions occurred. Likewise, *Betaentomopoxvirus* homologues were probably acquired from Group II *Alphabaculovirus* (Figure 4, yellow rectangle). Remarkably, ErelGV is the first *Betabaculovirus* with a *he65*-like gene (*ErelOrf-36* - Figure 4A, green rectangle) acquired from Group I *Alphabaculovirus*. It is not clear whether C-terminal deleted he65 remains functional in baculovirus. However the maintenance of the amino-terminal region indicates that this gene region is under positive selection pressure.

Furthermore, we found in ErelGV genome a *p43*-like gene (*ErelOrf-105*) whose homologues were found only in baculovirus species from the genus *Alphabaculovirus* (Figure 4B) with conserved amino acid sequence and position in the genome [7]. Deletion of *p43* in AcMNPV does not affect virus replication in cell culture and the reason for gene acquisition and preservation is not clear [57]. Two hypotheses can be raised for *p43* introduction in ErelGV: (*i*) ErelGV acquired the *p43*-like gene from Group I *Alphabaculovirus*, specifically from AcMNPV-related viruses; or (*ii*) ErelGV acquired from Group II *Alphabaculovirus*, specifically from a baculovirus (*e.g. Clanis bilineata nucleopolyhedrovirus -* ClbiNPV [58]) during co-infection of a sphingid host.

### Acquisitions of *Densovirus*-related genes in *Betabaculovirus*

**Figure 5:**
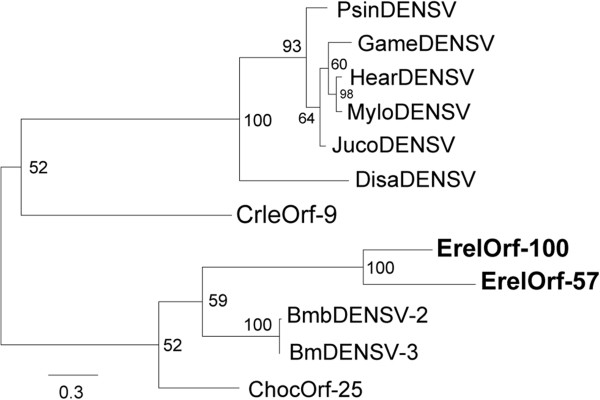
***Densovirus*** **-related genes in betabaculovirus and phylogenetic relationship.** ML tree was inferred using the alignment of ErelOrf-57 and ErelOrf-100 from ErelGV with non-structural protein (NS) from *Bombyx mori densovirus 2* and *3* (BmbDENSV-2 / Genbank YP_007714627.1 and BmDENSV-3/Genbank YP_007714627), NS3 from *Diatraea saccharalis densovirus* (DisaDENSV/Genbank NP_046812.1), *Mythimna loreyi densovirus* (MyloDENSV/Genbank NP_958098.1), *Helicoverpa armigera densovirus* (HaDENSV/Genbank AFK91982.1), *Galleria mellonella densovirus* (GameDENSV/Genbank NP_899649.1), *Junonia coenia densovirus* (JucoDENSV/Genbank AGO32182.1), and *Pseudoplusia includens densovirus* (PsinDENSV/Genbank YP_007003822.1), *Orf-25* from ChocGV, and *Orf-9* from CrleGV. The tree is midpoint rooted for purposes of clarity only. We hypothesized gene duplication for both ErelGV genes (boldface).

*ErelOrf-57* and *ErelOrf-100* are homologues to a non-structural *Densovirus* gene. *Densovirus*-related genes were previously described in two betabaculoviruses, ChocGV (*ChocOrf-25*) [21] and CrleGV (*CrleOrf-9*) [26], and one gammabaculovirus (baculovirus infective to hymenoptera), *Neodiprion lecontei nucleopolyhedrovirus* (NeleNPV *- NeleOrf-81*) [59]. The latter did not match the other two homologues (data not shown), suggesting these genes resulted from at least two HGT events between densoviruses and baculoviruses. Despite the limited number of *Densovirus* genomes available, we performed a phylogenetic analysis to help understand the origins of *Betabaculovirus* homologues. We found that the genes were dispersed over the phylogenetic tree, suggestive of multiple HGT events. As shown in Figure 5, *Betabaculovirus* homologues did not form a monophyletic cluster. To further substantiate our findings, we compared the likelihood of the observed tree to that estimated assuming a *Betabaculovirus* monophyletic clade (single-HGT event). Indeed, the likelihood ratio test rejected the monophyletic hypothesis favoring the multiple-HGT scenario, which was also supported by the distinct genomic context observed for the homologous betabaculovirus genes (data not shown). Moreover, both *ErelOrf-57* and *ErelOrf-100* form a well-supported clade, indicating that they probably represent a gene duplication event during ErelGV evolution.

## Conclusion

ErelGV is a new betabaculovirus species closely related to ChocGV and PiraGV isolates. Its genome encodes 130 ORFs, eight of which are unique. We found evidence suggesting horizontal gene transfers from *Alphabaculovirus* and *Densovirus* to *Betabaculovirus*. The *he65*-like gene was independently acquired tree times from *Alphabaculovirus*. We found a *dUTPase-like* gene homologous to OpMNPV *Orf-31* and two *Densovirus*-related genes. The contribution of these genes to baculovirus fitness is not clear and is being experimentally tested in our lab. Importantly, recombination events between insect viruses from unrelated families and genera might drive baculovirus genomic evolution.

### Nucleotide sequence accession number

The ErelGV genome sequence was submitted to GenBank under accession number KJ406702.

### Availability of supporting data

The complete ErelGV genome sequence has been submitted to GenBank (accession number KJ406702). All supporting data is included as additional files.

## Methods

### Virus purification

Insect cadavers of the hornworm *E. ello ello* with baculovirus infection symptoms were collected in cassava crops in the South of Brazil (Itajaí, Santa Catarina) in 1986. They were kindly provided by Dr. Renato Arcanjo Pegoraro (EPAGRI). The cadavers were kept in the freezer and later used for OBs purification. Insect cadavers were homogenized with ddH_2_O (w/v), filtered through three layers of gauze, and centrifuged at 7,000 *x g* for 10 min. The pellet was resuspended in 0.5% (w/v) SDS and again centrifuged at 7,000 *x* g for 10 min. The dilution and centrifugation steps were repeated four times, and the final pellet was washed in 0.5 M NaCl. The pellet was resuspended in ddH_2_O, loaded onto a continuous 40-65% sucrose gradient, and centrifuged at 104.000 *x g* for 40 min at 4°C. The OB band was collected, diluted 4-fold in ddH_2_O, and centrifuged at 7,000 *x g* for 15 min at 4°C.

### Electron microscopy

For scanning electron microscopy (SEM), 100 μl of the OB-containing solution (10^9^ OBs/ml) were incubated with 300 μl of acetone at 25°C for 1 hour. The solution was loaded in a metallic stub, dried overnight at 37°C, coated with gold in a Sputter Coater (Balzers) for 3 min, and observed in a scanning electron microscope Jeol JSM 840 A at 10 kV. For transmission electron microscopy (TEM) pellets of purified granules were fixed in Karnovsky fixative (2.5% glutaraldehyde, 2% paraformaldehyde, in 0.1 M, cacodylate buffer, pH 7.2) for 2 h, post-fixed in 1% osmium tetroxide in the same buffer for 1 h and then stained *en bloc* with 0.5% aqueous uranyl acetate, dehydrated in acetone, and embedded in Spurr’s low viscosity embedding medium. The ultrathin sections were contrasted with 2% uranyl acetate and observed in a ZEISS TEM 109 at 80 kV.

### Genomic DNA restriction analyses

Purified granules (10^9^ OBs/ml) were dissolved in an alkaline solution and used to extract DNA according to O’Reilly *et al*. [60]. The quantity and quality of the isolated DNA were determined by electrophoresis on 0.8% agarose (data not shown). The viral DNA (1–2 μg) was individually cleaved with the restriction enzymes *Hin*dIII, *EcoR*I, and *BamH*I (Promega) according to manufacturer’s instructions. The DNA fragments generated were analyzed by 0.8% agarose gel electrophoresis [61], visualized, and photographed in AlphaImager® Mini (Alpha Innothech).

### Genome sequencing, assembly, and annotation

ErelGV genomic DNA was sequenced with the 454 Genome Sequencer (GS) FLX™ Standard (Roche) at the Centro de Genômica de Alto Desempenho do Distrito Federal (Brasília, Brazil). The genome was assembled *de novo* using Geneious 6.0 [62] and confirmed using restriction enzyme digestion profile. The annotation was performed using Geneious 6.0 to identify the open reading frames (ORFs) that started with a methionine codon (ATG) encoding at least 50 amino acids and blastp [63] to identify homologues.

### Phylogeny, genome, and gene comparisons

For *Baculoviridae* phylogenetic analysis, a MAFFT alignment [64] was carried out with concatenated amino acid sequences predicted for 37 baculovirus core genes. A maximum likelihood tree was inferred using PhyML with 100 repetitions of a non parametric bootstrap [65], implemented in Geneious, with LG + I + G + F model selected by Prottest 2.4 [66]. Moreover, a genomic comparison was performed using the protein dataset of all the complete *Betabaculovirus* genomes available in Genbank. The dataset was compared using CGView Comparison Tool [36] and the results were plotted using CIRCOS [37]. We also compared ChocGV and PiraGV genomes with ErelGV genome using Mauve alignment [34]. The horizontal gene transfer (HGTs) events were investigated comparing the maximum likelihood phylogenetic tree inferred using the RAxML method [67] and a MAFT alignment of homologues for *he65*-like and *p43*-like, and *Densovirus*-related genes with 100 repetitions of a non parametric bootstrap for branch support.

## Electronic supplementary material

Additional file 1: Table S1: Characteristics of the *Erinnyis ello granulovirus* (ErelGV) genome: analysis and homology search. Predicted ORFs are compared with homologous genes in three related genomes. (DOCX 67 KB)
