# Supplementary material for: Genome sequence of Erinnyis ello granulovirus (ErelGV), a natural cassava hornworm pesticide and the first sequenced sphingid-infecting betabaculovirus
Source: BMC Genomics. 2014 Oct 4;15(1):856. doi: 10.1186/1471-2164-15-856 (PMC4192325; doi:10.1186/1471-2164-15-856)
Supplement: Supplementary file 1 — Additional file 1: Table S1: Characteristics of the Erinnyis ello granulovirus (ErelGV) genome: analysis and homology search. Predicted ORFs are compared with homologous genes in three related genomes. (DOCX 67 KB) [file 12864_2014_6515_MOESM1_ESM.docx]

| **Table S1**. Characteristics of the *Erinnyis ello granulovirus* (ErelGV) genome: analysis and homology search. Predicted ORFs are compared with homologous genes in three related genomes. | | | | | | | | | | | | | | | | |
| --- | --- | --- | --- | --- | --- | --- | --- | --- | --- | --- | --- | --- | --- | --- | --- | --- |
| **Orf** | **Name** | **Promoter**  **motif** | **Position** | | | **Size**  **(nt)** | **Size (aa)** |  | **CypoGV** | |  | **ChocGV** | |  | **PiraGV** | |
|  |  |  |  |  |  |  |  |  | **ORF** | **Max Id (%)** |  | **ORF** | **Max Id (%)** |  | **ORF** | **Max Id (%)** |
| 1^b^ | *granulin* | L | 1 | > | 747 | 747 | 248 |  | 1 | 96.40 |  | 1 | 97.20 |  | 1 | 87.50 |
| 2 |  | L | 744 | < | 1,082 | 339 | 112 |  | 2 | 51.60 |  | 2 | 50.00 |  | 2 | 60.30 |
| 3 ^c^ | *pk-1* | E | 1,063 | > | 1,899 | 837 | 278 |  | 3 | 57.00 |  | 3 | 65.80 |  | 3 | 67.80 |
| 4 |  | ? | 1,996 | > | 2,541 | 546 | 181 |  | - | - |  | - | - |  | - | - |
| 5 | *dUTPase*-like | E, L | 2,811 | > | 3,764 | 954 | 317 |  | 16^e^ | 31.30 |  | - | - |  | - | - |
| 6 |  | E | 3,839 | < | 4,414 | 576 | 191 |  | 4 | 50.30 |  | 5 | 53.40 |  | 4 | 58.50 |
| 7 |  | ? | 4,404 | > | 4,643 | 240 | 79 |  | 5 | 45.20 |  | 6 | 53.30 |  | 5 | 57.90 |
| 8 ^c^ | *ie-1* | E | 4,743 | < | 6,059 | 1,317 | 438 |  | 7 | 45.10 |  | 7 | 54.70 |  | 6 | 56.30 |
| 9 ^c^ |  | ? | 6,090 | > | 6,665 | 576 | 191 |  | 8 | 44.70 |  | 8 | 51.50 |  | 7 | 48.40 |
| 10 ^b^ |  | ? | 6,693 | < | 6,998 | 306 | 101 |  | 9 | 65.30 |  | 9 | 66.30 |  | 8 | 64.40 |
|  | *hr1* |  | 7,113 | - | 7,204 | 92 |  |  |  |  |  |  |  |  |  |  |
| 11* |  | ? | 7,151 | < | 7,792 | 642 | 213 |  | - | - |  | - | - |  | - | - |
| 12 |  | E | 7,791 | > | 7,949 | 159 | 52 |  | - | - |  | - | - |  | - | - |
| 13 ^a^ | *odv-e18* | L | 8,172 | < | 8,447 | 276 | 91 |  | 14 | 73.60 |  | 12 | 79.50 |  | 14 | 69.40 |
| 14 ^a^ | *p49* | E, L | 8,448 | < | 9,827 | 1,380 | 459 |  | 15 | 56.20 |  | 13 | 60.60 |  | 15 | 61.10 |
| 15* |  | E | 9,736 | > | 9,960 | 225 | 74 |  | - | - |  | - | - |  | - | - |
| 16 ^a^ | *odv-e56/pif-5* | L | 9,975 | < | 11,033 | 1,059 | 352 |  | 18 | 69.50 |  | 14 | 73.40 |  | 16 | 67.20 |
| 17 |  | E, L | 11,051 | < | 11,530 | 480 | 159 |  | - | - |  | - | - |  | 17 | 29.30 |
| 18 |  | E, L | 11,545 | < | 11,943 | 399 | 132 |  | - | - |  | - | - |  | 18 | 35.40 |
| 19 |  | E | 12,013 | > | 12,195 | 183 | 60 |  | 19 | 40.70 |  | 16 | 41.80 |  | 19 | 47.30 |
|  | *hr2* |  | 12,183 | - | 12,222 | 40 |  |  |  |  |  |  |  |  |  |  |
| 20 | *pep-1* | E, L | 12,238 | < | 12,765 | 528 | 175 |  | 20 | 58.90 |  | 17 | 71.30 |  | 20 | 54.00 |
| 21 | *pep/p10* | E, L | 12,885 | > | 13,910 | 1,026 | 341 |  | 22 | 69.20 |  | 18 | 66.40 |  | 21 | 67.60 |
| 22 ^b^ | *pep-2* | E, L | 13,945 | > | 14,397 | 453 | 150 |  | 23 | 62.20 |  | 19 | 62.80 |  | 22 | 59.90 |
| 23 |  | ? | 14,494 | < | 15,636 | 1,143 | 380 |  | 27 | 24.30 |  | - | - |  | - | - |
|  | *hr3* |  | 15,715 | - | 15,853 | 139 |  |  |  |  |  |  |  |  |  |  |
|  | *hr4* |  | 15,950 | - | 16,023 | 74 |  |  |  |  |  |  |  |  |  |  |
| 24 |  | E | 16,087 | < | 16,248 | 162 | 53 |  | - | - |  | - | - |  | - | - |
| 25 |  | E | 16,439 | > | 16,723 | 285 | 94 |  | - | - |  | - | - |  | - | - |
| 26 |  | E | 16,647 | > | 17,879 | 1,233 | 410 |  | - | - |  | 22 | 48.20 |  | 24 | 31.00 |
| 27* |  | E | 17,928 | < | 18,116 | 189 | 62 |  | - | - |  | - | - |  | - |  |
| 28^d^ | *f protein* | E | 18,356 | > | 20,137 | 1,782 | 593 |  | 31 | 56.60 |  | 23 | 60.90 |  | 27 | 58.70 |
| 29 |  | E | 20,368 | > | 21,297 | 930 | 309 |  | - | - |  | - | - |  | - | - |
| 30 |  | E, L | 21,359 | < | 22,069 | 711 | 236 |  | 33 | 31.30 |  | 24 | 44.00 |  | 28 | 43.60 |
| 31 ^a^ | *pif-3* | E, L | 22,107 | > | 22,673 | 567 | 188 |  | 35 | 52.90 |  | 26 | 47.80 |  | 30 | 54.10 |
| 32 |  | E, L | 22,695 | > | 23,006 | 312 | 103 |  | 39 | 62.10 |  | 28 | 61.40 |  | 31 | 55.30 |
| 33 |  | L | 23,038 | < | 23,352 | 315 | 104 |  | 40 | 34.70 |  | - | - |  | - | - |
| 34 ^a^ | *lef-2* | ? | 23,501 | > | 24,028 | 528 | 175 |  | 41 | 49.10 |  | 29 | 54.40 |  | 33 | 55.00 |
| 35 |  | E | 24,012 | > | 24,284 | 273 | 90 |  | 42 | 41.90 |  | 30 | 43.70 |  | 34 | 44.20 |
| 36 | *he65*-like | L | 24,247 | < | 25,026 | 780 | 259 |  | - | - |  | - | - |  | - | - |
| 37 |  | E | 25,007 | < | 25,345 | 339 | 112 |  | 43 | 29.40 |  | 31 | 38.70 |  | 35 | 37.00 |
| 38 |  | E, L | 25,355 | < | 25,801 | 447 | 148 |  | 45 | 36.30 |  | 32 | 56.60 |  | 36 | 56.30 |
| 39 | *mp-nase* | E, L | 25,859 | < | 27,247 | 1,389 | 462 |  | 46 | 39.70 |  | 33 | 43.80 |  | 37 | 46.50 |
| 40 | *p13* | L | 27,226 | > | 28,071 | 846 | 281 |  | 47 | 63.40 |  | 34 | 65.50 |  | 38 | 58.70 |
| 41 | *chtBP* | L | 28,091 | > | 28,348 | 258 | 85 |  | 9 | 22.50 |  | 7 | 23.90 |  | 8 | 25.30 |
| 42 ^a^ | *pif-2* | L | 28,358 | > | 29,482 | 1,125 | 374 |  | 48 | 70.40 |  | 35 | 69.00 |  | 40 | 69.20 |
| 43 | *pp-1* | L | 29,489 | > | 29,779 | 291 | 96 |  | - | - |  | 36 | 45.30 |  | - | - |
| 44 |  | L | 29,694 | > | 32,642 | 2,949 | 982 |  | 50 | 37.40 |  | - | - |  | - | - |
| 45 ^b^ |  | L | 32,639 | < | 33,292 | 654 | 217 |  | 52 | 78.00 |  | 37 | 89.00 |  | 43 | 72.00 |
| 46 ^c^ |  | L | 33,302 | > | 33,454 | 153 | 50 |  | 53 | 63.80 |  | 38 | 70.00 |  | 44 | 57.00 |
| 47 ^c^ | *v-ubq* | E | 33,462 | < | 33,749 | 288 | 95 |  | 54 | 82.10 |  | 39 | 87.20 |  | 45 | 85.30 |
| 48 ^a^ |  | L | 33,841 | > | 34,896 | 1,056 | 351 |  | 55 | 52.30 |  | 40 | 66.20 |  | 46 | 63.60 |
| 49 ^b^ |  | E | 34,794 | > | 35,066 | 273 | 90 |  | 56 | 57.40 |  | 41 | 54.30 |  | 47 | 57.40 |
| 50 ^c^ | *39k; pp31* | E | 35,067 | < | 35,903 | 837 | 278 |  | 57 | 40.00 |  | 42 | 55.70 |  | 48 | 58.90 |
| 51 ^b^ | *lef-11* | L | 35,884 | < | 36,177 | 294 | 97 |  | 58 | 53.30 |  | 43 | 52.20 |  | 49 | 57.40 |
| 52 | *sod* | L | 36,213 | < | 36,686 | 474 | 157 |  | 59 | 65.90 |  | 44 | 70.70 |  | 50 | 68.80 |
| 53* |  | ? | 36,615 | > | 36,806 | 192 | 63 |  | - | - |  | - | - |  | - | 45.80 |
|  | *hr5* |  | 36,954 | - | 37,144 | 191 |  |  |  |  |  |  |  |  |  |  |
| 54 | *p10* | E, L | 36,966 | < | 37,331 | 366 | 121 |  | - | - |  | 45 | 54.50 |  | - | - |
| 55 ^a^ | *p74* | E, L | 37,344 | < | 39,308 | 1,965 | 654 |  | 60 | 60.10 |  | 46 | 62.70 |  | 51 | 58.80 |
| 56 |  | L | 39,312 | < | 39,689 | 378 | 125 |  | - | - |  | 53 | 29.50 |  | - | 34.70 |
| 57 |  | E | 39,763 | < | 40,482 | 720 | 239 |  | - | - |  | 25 | 35.10 |  | - | - |
| 58 |  | E | 40,640 | < | 41,245 | 606 | 201 |  | - | - |  | 48 | 61.10 |  | 54 | 61.20 |
| 59* |  | L | 41,405 | < | 41,578 | 174 | 57 |  | - | - |  | - | - |  | - | - |
| 60 |  | L | 41,599 | < | 41,868 | 270 | 89 |  | 62 | 76.20 |  | 49 | 79.60 |  | 55 | 71.00 |
| 61 ^a^ | *p47* | E, L | 41,943 | > | 43,124 | 1,182 | 393 |  | 68 | 65.50 |  | 50 | 66.70 |  | 56 | 66.30 |
| 62 ^c^ | *bv-e31* | E, L | 43,170 | > | 43,835 | 666 | 221 |  | 69 | 66.50 |  | 51 | 67.60 |  | 57 | 67.00 |
| 63 ^c^ | *p24* | L | 43,849 | > | 44,412 | 564 | 187 |  | 71 | 62.50 |  | 52 | 67.80 |  | 58 | 67.70 |
| 64 |  | ? | 44,409 | > | 44,696 | 288 | 95 |  | - | - |  | 53 | 45.50 |  | - | - |
| 65 ^c^ | *38.7k* | ? | 44,748 | < | 45,221 | 474 | 157 |  | 73 | 27.40 |  | 54 | 34.80 |  | 59 | 31.50 |
| 66 ^a^ | *lef-1* | ? | 45,202 | < | 45,906 | 705 | 234 |  | 74 | 57.00 |  | 55 | 64.70 |  | 60 | 63.40 |
| 67 ^a^ | *pif-1* | L | 45,937 | > | 47,547 | 1,611 | 536 |  | 75 | 65.50 |  | 56 | 67.20 |  | 61 | 58.70 |
| 68 | *fgf-1* | ? | 47,548 | < | 48,231 | 684 | 227 |  | 76 | 45.60 |  | 57 | 55.30 |  | 62 | 54.90 |
| 69 |  | E | 48,295 | < | 48,615 | 321 | 106 |  | 77 | 37.40 |  | 58 | 50.60 |  | 63 | 44.20 |
| 70* |  | E, L | 48,620 | > | 48,772 | 153 | 50 |  | - | - |  | - | - |  | - | - |
| 71 |  | E, L | 48,785 | > | 49,291 | 507 | 168 |  | 79 | 36.30 |  | 59 | 39.30 |  | 64 | 41.30 |
| 72 ^c^ | *lef-6* | E | 49,262 | < | 49,564 | 303 | 100 |  | 80 | 38.00 |  | 60 | 38.80 |  | 65 | 50.00 |
| 73 ^b^ | *dbp* | E | 49,649 | < | 50,494 | 846 | 281 |  | 81 | 48.80 |  | 61 | 64.70 |  | 66 | 60.60 |
| 74 |  | L | 50,509 | < | 50,739 | 231 | 76 |  | 82 | 45.80 |  | 62 | 52.90 |  |  |  |
| 75 |  | E | 50,675 | < | 51,244 | 570 | 189 |  | 82 | 31.70 |  | 63 | 38.50 |  | 67 | 41.90 |
| 76 ^a^ | *p48* | E, L | 51,268 | > | 52,440 | 1,173 | 390 |  | 83 | 73.20 |  | 64 | 74.80 |  | 68 | 75.10 |
| 77 ^c^ |  | E, L | 52,482 | > | 52,811 | 330 | 109 |  | 84 | 57.40 |  | 65 | 68.40 |  | 69 | 57.40 |
| 78 ^a^ |  | L | 52,865 | > | 53,989 | 1,125 | 374 |  | 85 | 63.50 |  | 66 | 71.30 |  | 70 | 70.20 |
| 79 ^a^ | *p6.9* | E, L | 54,035 | > | 54,214 | 180 | 59 |  | 86 | 49.10 ^e^ |  | 67 | 67.80 ^e^ |  | 71 | 69.40 ^e^ |
| 80 ^a^ | *lef-5* | ? | 54,264 | < | 54,995 | 732 | 243 |  | 87 | 64.20 |  | 68 | 66.90 |  | 72 | 64.20 |
| 81 ^a^ | *38K* | L | 54,942 | > | 55,841 | 900 | 299 |  | 88 | 56.10 |  | 69 | 66.00 |  | 73 | 63.50 |
| 82 ^a^ | *odv-e28/pif-4* | L | 55,838 | < | 56,323 | 486 | 161 |  | 89 | 56.90 |  | 70 | 68.10 |  | 74 | 65.60 |
| 83 ^a^ | *dna-helicase-1* | L | 56,389 | > | 59,694 | 3,306 | 1101 |  | 90 | 53.00 |  | 71 | 71.30 |  | 75 | 64.30 |
| 84 ^a^ | *odv-e25* | E, L | 59,724 | < | 60,365 | 642 | 213 |  | 91 | 77.90 |  | 72 | 77.80 |  | 76 | 76.60 |
| 85 ^a^ |  | E, L | 60,389 | < | 60,877 | 489 | 162 |  | 92 | 50.90 |  | 73 | 50.90 |  | 77 | 57.50 |
| 86 ^a^ | *p33/sox* | L | 60,914 | > | 61,678 | 765 | 254 |  | 93 | 63.40 |  | 74 | 66.90 |  | 78 | 68.00 |
| 87 ^a^ | *lef-4* | E, L | 61,675 | < | 63,042 | 1,368 | 455 |  | 95 | 54.10 |  | 75 | 62.10 |  | 80 | 55.90 |
| 88 ^a^ | *vp39* | L | 63,119 | > | 63,979 | 861 | 286 |  | 96 | 57.80 |  | 76 | 63.40 |  | 81 | 64.50 |
| 89 ^a^ | *odv-e27* | ? | 64,040 | > | 64,894 | 903 | 300 |  | 97 | 61.50 |  | 77 | 73.10 |  | 82 | 64.20 |
|  | *hr6* |  | 64,964 | - | 65,177 | 214 |  |  |  |  |  |  |  |  |  |  |
| 90* |  | L | 65,057 | > | 65,233 | 177 | 58 |  | - | - |  | - | - |  | - | - |
| 91 |  | E, L | 65,159 | < | 66,259 | 1,101 | 366 |  | 90 | 40.20 |  | 78 | 45.00 |  | 83 | 41.40 |
| 92 |  | ? | 66,258 | > | 66,632 | 375 | 124 |  | 91 | 48.10 |  | 79 | 40.90 |  | 84 | 41.90 |
| 93 ^a^ | *p95/vp91* | E, L | 66,619 | < | 68,409 | 1,791 | 596 |  | 92 | 43.00 |  | 80 | 54.50 |  | 85 | 41.80 |
| 94 ^c^ |  | L | 68,399 | > | 68,806 | 408 | 135 |  | 102 | 50.00 |  | 81 | 31.70 |  | 86 | 34.10 |
| 95 ^a^ |  | E, L | 68,784 | > | 69,365 | 582 | 193 |  | 94 | 67.60 |  | 82 | 72.50 |  | 87 | 67.90 |
| 96 ^a^ | *gp41* | E, L | 69,343 | > | 70,179 | 837 | 278 |  | 95 | 62.60 |  | 83 | 66.10 |  | 88 | 63.50 |
| 97 | *iap-3* | E, L | 70,213 | > | 71,022 | 810 | 269 |  | 17 | 43.40 |  | 84 | 42.30 |  | 79 | 32.40 |
| 98 ^a^ |  | ? | 71,032 | > | 71,322 | 291 | 96 |  | 105 | 39.30 |  | 85 | 46.60 |  | 89 | 39.80 |
| 99 ^a^ | *vlf-1* | L | 71,246 | > | 72,367 | 1,122 | 373 |  | 106 | 68.80 |  | 86 | 65.80 |  | 90 | 70.80 |
| 100 |  | E, L | 72,429 | < | 73,097 | 669 | 222 |  | - | - |  | 25 | 25.20 |  | - | - |
| 101 |  | E | 73,141 | < | 73,818 | 678 | 225 |  | - | - |  | 87 | 43.50 |  | - | - |
| 102* |  | ? | 73,812 | > | 73,976 | 165 | 54 |  | - | - |  | - | - |  | - | - |
| 103 |  | E, L | 73,909 | > | 74,112 | 204 | 67 |  | 107 | 59.40 |  | 88 | 71.20 |  | 91 | 67.30 |
| 104 ^b^ |  | E, L | 74,174 | > | 74,626 | 453 | 150 |  | 108 | 58.70 |  | 89 | 64.00 |  | 92 | 63.30 |
| 105 | *p43*-like | ? | 74,618 | < | 75,706 | 1,089 | 362 |  | - | - |  | - | - |  | - | - |
| 106 ^a^ | *dna-pol* | E, L | 75,743 | < | 78,901 | 3,159 | 1052 |  | 111 | 62.30 |  | 90 | 68.70 |  | 93 | 67.10 |
| 107 ^a^ | *desmoplakin* | ? | 78,849 | > | 80,942 | 2,094 | 697 |  | 112 | 34.70 |  | 91 | 35.40 |  | 94 | 38.50 |
| 108 ^c^ | *lef-3* | E | 81,064 | < | 82,077 | 1,014 | 337 |  | 113 | 41.40 |  | 92 | 60.50 |  | 95 | 52.50 |
| 109 ^a^ | *pif-6* | ? | 82,049 | > | 82,426 | 378 | 125 |  | 114 | 56.10 |  | 93 | 65.60 |  | 96 | 66.40 |
| 110 |  | ? | 82,477 | > | 83,064 | 588 | 195 |  | 115 | 31.40 |  | 94 | 43.60 |  | 97 | 48.30 |
| 111 | *iap-5* | E | 83,045 | > | 83,887 | 843 | 280 |  | 116 | 48.90 |  | 95 | 56.00 |  | 98 | 53.40 |
| 112 ^a^ | *lef-9* | ? | 83,865 | > | 85,346 | 1,482 | 493 |  | 117 | 69.40 |  | 96 | 73.40 |  | 99 | 73.60 |
| 113 ^b^ | *fp-25k* | E, L | 85,352 | > | 85,816 | 465 | 154 |  | 118 | 65.20 |  | 97 | 70.80 |  | 100 | 63.60 |
| 114 | *dna-ligase* | ? | 85,813 | < | 87,486 | 1,674 | 557 |  | 120 | 60.10 |  | 99 | 66.80 |  | 102 | 65.70 |
| 115 |  | ? | 87,658 | > | 87,888 | 231 | 76 |  | 121 | 45.00 |  | 100 | 43.60 |  | 103 | 39.60 |
| 116 |  | L | 87,943 | > | 88,161 | 219 | 72 |  | 122 | 63.00 |  | 101 | 60.30 |  | 104 | 55.10 |
| 117 | *fgf-2* | ? | 88,222 | < | 89,418 | 1,197 | 398 |  | 123 | 34.30 |  | 102 | 34.50 |  | 105 | 34.20 |
| 118 |  | E, L | 89,545 | > | 89,823 | 279 | 92 |  | 124 | 52.80 |  | 103 | 60.70 |  | 106 | 61.10 |
| 119 ^a^ | *alk-exo* | E | 89,873 | > | 91,078 | 1,206 | 401 |  | 125 | 56.30 |  | 104 | 63.90 |  | 107 | 62.50 |
| 120 | *dna-helicase-2* | E | 90,987 | > | 92,357 | 1,371 | 456 |  | 126 | 54.90 |  | 105 | 58.90 |  | 108 | 54.60 |
| 121 |  | ? | 92,402 | < | 93,457 | 1056 | 351 |  | 130 | 41.20 |  | 106 | 41.00 |  | 109 | 32.90 |
|  | *hr7* |  | 92,550 | - | 92,622 | 73 |  |  |  |  |  |  |  |  |  |  |
| 122 ^a^ | *lef-8* | E, L | 93,472 | < | 96,081 | 2,610 | 869 |  | 131 | 70.50 |  | 107 | 73.50 |  | 110 | 71.40 |
| 123 ^a^ |  | L | 96,357 | > | 96,755 | 399 | 132 |  | 134 | 60.20 |  | 109 | 60.20 |  | 113 | 65.40 |
| 124 |  | L | 96,752 | < | 97,546 | 795 | 264 |  | 135 | 35.60 |  | 110 | 43.20 |  | 114 | 42.90 |
| 125 | *lef-10* | E | 97,773 | > | 98,159 | 387 | 128 |  | 137 | 51.40 |  | 112 | 61.60 |  | 115 | 56.00 |
| 126 ^a^ | *vp1054* | ? | 98,026 | > | 99,036 | 1,011 | 336 |  | 138 | 57.40 |  | 113 | 63.80 |  | 116 | 64.30 |
| 127 |  | L | 99,033 | > | 99,206 | 174 | 57 |  | - | - |  | - | - |  | 117 | 42.10 |
| 128 | *fgf-3* | E | 99,231 | > | 100,130 | 900 | 299 |  | 140 | 35.90 |  | 114 | 40.20 |  | 118 | 39.80 |
| 129 | *egt* | E | 100,150 | < | 101,550 | 1,401 | 466 |  | 141 | 48.90 |  | 115 | 52.30 |  | 119 | 50.30 |
| 130^c^ | *me53* | E | 101,729 | > | 102,709 | 981 | 326 |  | 143 | 49.00 |  | 116 | 47.00 |  | 120 | 50.20 |
|  | | | | | | | | | | | | | | | | |
| ^a^ α-, β-, γ-, and δ-baculovirus core genes; ^b^ α-, β-, and γ-baculovirus core genes; ^c^ α- and β-baculovirus core genes; ^d^ α-, β-, and δ-baculovirus core genes; ^e^ Identity was achieved by manual alignment. * ErelGV unique genes. The putative gene upstream regions were classified according to the presence of promoter motifs in early (E), late (L), or unknown (?). | | | | | | | | | | | | | | | | |
